# Development of Adaptive Communication Skills in Infants of Blind Parents

**DOI:** 10.1037/dev0000564

**Published:** 2018-10-18

**Authors:** Nataşa Ganea, Kristelle Hudry, Angélina Vernetti, Leslie Tucker, Tony Charman, Mark H. Johnson, Atsushi Senju

**Affiliations:** 1 Centre for Brain and Cognitive Development, Birkbeck, University of London; 2 Victorian Autism Specific Early Learning and Care Centre and Olga Tennison Autism Research Centre, School of Psychology and Public Health, La Trobe University; 3 Centre for Brain and Cognitive Development, Birkbeck, University of London; 4 Department of Psychology, Institute of Psychiatry, Psychology and Neuroscience, King’s College London; 5 Centre for Brain and Cognitive Development, Birkbeck, University of London

**Keywords:** development, communication, interaction, infants, blind parents

## Abstract

A fundamental question about the development of communication behavior in early life is how infants acquire adaptive communication behavior that is well-suited to their individual social environment, and how the experience of parent-child communication affects this development. The current study investigated how infants develop communication skills when their parents are visually impaired and cannot see their infants’ eye gaze. We analyzed 6-min video recordings of naturalistic interaction between 14 sighted infants of blind parents (SIBP) with (a) their blind parent, and (b) a sighted experimenter. Data coded from these interactions were compared with those from 28 age-matched sighted infants of sighted parents (controls). Each infant completed two visits, at 6–10 months and 12–16 months of age. Within each interaction sample, we coded the function (initiation or response) and form (face gaze, vocalization, or action) of each infant communication behavior. When interacting with their parents, SIBP made relatively more communicative responses than initiations, and used more face gaze and fewer actions to communicate, than did controls. When interacting with a sighted experimenter, by contrast, SIBP made slightly (but significantly) more communicative initiations than controls, but otherwise used similar forms of communication. The differential communication behavior by infants of blind versus sighted parents was already apparent by 6–10 months of age, and was specific to communication with the parent. These results highlight the flexibility in the early development of human communication behavior, which enables infants to optimize their communicative bids and methods to their unique social environment.

Communication is a cognitive skill manifest through complex social behavior that consists of sending information to and receiving information from another (Jaswal & Fernald, 2002), and forms a fundamental part of human social interaction and social learning. From very early in postnatal development, infants use a wide range of channels to communicate with adults. Infants detect and preferentially look at faces that make eye contact (Farroni, Csibra, Simion, & Johnson, 2002), recognize and respond to their mother’s voice (DeCasper & Fifer, 1980), and use information about their own goal-directed actions to detect goals in others’ actions (Sommerville, Woodward, & Needham, 2005). All of these channels allow infants to receive communicative information from, and send signals to, adults from the first days of life.

Research has demonstrated that infants actively exploit these channels to initiate and respond to communication with adults. A clear example of infants’ initiation of communication is in their object-directed action which has been shown to attract parents’ attention, with parents being more likely to follow their infants’ interest and explore the objects themselves as well as to use more referential language (Tamis-LeMonda, Kuchirko, & Tafuro, 2013). By contrast, infants respond to adults’ communicative acts by looking toward them and attending to their actions. This behavior is thought to set the foundation for referential communication (Bakeman & Adamson, 1984), and has been found to be reduced in 12-month-old infants who are later diagnosed with autism spectrum disorder (ASD; Wan et al., 2013), a condition characterized by core social-communication impairment, alongside behavioral inflexibility.

Reciprocal sensitivity to each partner’s vocalizations is also reported within parent-child interactions, from infancy. For example, from at least five and a half months of age, infants respond contingently to their mothers’ vocalizations (Bornstein, Putnick, Cote, Haynes, & Suwalsky, 2015) and, in turn, infants’ vocalizations engage the parents who are more likely to vocalize back to the infants (Goldstein & West, 1999; Gros-Louis, West, & King, 2016). This research points to the fact that infants’ communication behavior is closely linked to that of their communicative partners, and that infants play an active role when communicating with adults.

A fundamental question about the development of communication behavior is how infants acquire these skills, and how the experience of parent-child communication affects their development. The study of sighted infants of blind parents (SIBP) provides an intriguing opportunity for elucidating typical developmental processes, because this group of infants will not experience immediate responses from parents that are contingent upon visual modes of communication—such as eye contact, or gestures/actions which involve no physical contact—because their parents cannot see them. Given the major role that forms of communication specific to the visual channel—such as eye gaze (Corkum & Moore, 1995) and gestures (Csibra, 2003)—play in the typical development of early parent-infant communication, and the broad downstream effects of an altered developmental experience for children with congenital visual impairment (e.g., Tadić, Pring, & Dale, 2009), it is crucial to investigate the development of communication skills among SIBP.

To date, only a handful of studies have reported on the communication skills of SIBP, possibly due to the difficulty in accessing the target population. Early qualitative research, often involving in-depth follow up of a small sample, has consistently reported that parental visual impairment has very little impact on the overall quality of parent-child communication which seems to be adaptable via different channels, such as through auditory and tactile communication behaviors. In the first single case study of a sighted infant of two blind parents, Adamson, Als, Tronick, and Brazelton (1977) found that the infant looked less at her mother—who also showed less modulation of her own facial expressions—but was very engaged with her father—whose actions she followed closely. When questioned about his ability to monitor his infant’s attention, the father reported that he used the direction of her breath as a cue to judge whether or not she was looking at him. By contrast, the mother reported that she tended to rely more on touch to monitor her infant’s attention, which proved distracting for the infant, especially during feeding.

Another qualitative study of four SIBP (Collis & Bryant, 1981) similarly indicated that blind parents relied more on language and touch to engage with their children. In particular, these parents exploited distinctive sounds made by objects in the room to monitor their child’s location and, during periods of silence, they checked in verbally by calling the child’s name, making remarks or comments about the child, or asking the child to bring them an object. Each of these behaviors provided opportunities to locate the child but also to engage in interaction when the child responded. Rattray and Zeedyk (2005) quantified the communication behavior of five parent–child dyads affected by visual impairment on behalf of either the parent *and*/*or* the child and reported that all dyads relied on touch, vocalization and facial orientation to maintain communicative interaction.

Recently, efforts have been made to quantify the communication behavior of SIBP, including studies comparing groups of SIBP with control groups of infants with sighted parents (hereafter, controls). Senju et al. (2013) reported the first such study, looking at the forms of communication used by a small number of SIBP (*n* = 5) during free play interaction with their blind parent. Similar to the qualitative/single case study reports presented above, Senju et al. (2013) found no differences in the overall quantity of communication behavior between SIBP and controls. However, SIBP vocalized more than controls, and tended to look less at their parents, although this latter difference did not reach statistical significance. Chiesa, Galati, and Schmidt (2015) also recently compared the communication behaviors of seven SIBP (aged from 6 months to 3 years) to those of seven age- and gender-matched controls, replicating Senju et al.’s (2013) finding that SIBP looked less frequently at their parents and vocalized more during interaction than did controls. These studies corroborate the earlier qualitative accounts, suggesting a typical range of overall communication behaviors among SIBP, compared with controls, albeit with possible differences in the specific channels of communication used by SIBP for interaction with their blind caregivers.

There are at least two contrasting theoretical viewpoints that can account for the suggestion that interacting with a blind parent may influence certain aspects of communication behavior in infants, without broadly impairing development in this domain. The affective learning model (Dawson, Webb, & McPartland, 2005; Grelotti, Gauthier, & Schultz, 2002) emphasizes the role of the reward value of communication behavior that could emerge as a result of extensive exposure to the co-occurrence of communication behavior and a wide variety of positive experiences through social interaction and communication. From this position, SIBP could fail to develop the usual expertise and interest in adults’ gaze because their own use and processing of gaze is not reciprocated by their blind parent, and therefore does not become rewarding. (This is compared with auditory or tactile forms of communication which should be reciprocated equally—or to even greater extent—among SIBP and their parents, than among control dyads). Alternatively, the interactive specialization model (Johnson, 2011) assumes that infants are born with widespread connections between cortical and subcortical regions of the brain (Elman et al., 1996) and that input from subcortical routes interacts with architectural biases to form specialized networks for social cognition. This model of developing brain functions predicts that SIBP could develop different forms of specialized communication behaviors, optimized to fit adaptively with the unique input and contingent responses provided by their blind parents.

In light of these perspectives, the current study aimed to compare communicative behaviors across matched groups of SIBP and control infants, elicited during naturalistic social interaction scenarios—parent-child interaction (PCI), and interaction between the child and an unfamiliar sighted adult (i.e., stranger-child interaction [SCI]). The affective learning viewpoint would predict that the differences in communication behavior between SIBP and controls should not be limited to PCI but generalize to SCI, because communication behavior is based on the passively learned reward value of such behavior, primarily through interaction with the blind primary caregiver. By contrast, the interactive specialization model would predict that the communication behavior of SIBP could manifest differently between PCI and SCI conditions, because this has developed as an active adaptation to optimize communication with the blind primary caregiver, which should generate different dynamics of interaction when they communicate with other sighted adults.

To quantify infant communication behaviors, we adopted a coding scheme initially developed by Clifford, Hudry, Brown, Pasco, and Charman (2010), whereby each identified child communication act is assigned a code for function (i.e., initiation vs. response) and one or more forms (i.e., face gaze, vocalization, and gesture/action). In this way, we captured both the *pragmatic context* in which successful communication behaviors occurred (i.e., the function of communication acts), and the specific ways in which the infants communicated with their social partners (i.e., the form/s used to convey communication acts). Both of these aspects of communication were coded, as similar forms of communication (e.g., looking at the partner while vocalizing) could denote either a communication episode that the infant initiated (e.g., when seeking help from the partner to get an object that is out of reach), or one occurring in response to the adult (e.g., labeling an object held up by the adult). To capture any developmental changes in communication, we included a prospective follow up within our design which allowed us to investigate the patterns of communication behavior between groups and across communication contexts, during the latter half of the first year of life and the first half of the second year of life.

## Method

### Design and Participants

We employed a 2 (group: SIBP vs. control) × 2 (timepoint) × 2 (communication context: PCI vs. SCI) mixed between-within subjects design, with infants filmed playing with their mothers (PCI) and with an unfamiliar, sighted female researcher (SCI) at each visit. These data represent secondary analysis of a dataset already reported by Senju et al. (2015), a subsample of which (*n* = 5 SIBP) have previously been reported by Senju et al. (2013). The procedure was approved by the Research Ethics Committee of Department of Psychological Sciences, Birkbeck, University of London (Project title: Cognitive Development of Sighted Infants of Blind Parents; Protocol no.: 7842).

Our SIBP group comprised 14 parent-infant dyads, recruited via charities and parental support groups relevant to blind adults, and personal contacts. These dyads included sighted infants (7 female)—Aged 6–10 months at Time 1 (*M* = 8.85, *SD* = 1.10) and 12–16 months at Time 2 (*M* = 14.28, *SD* = 0.88), with mean between-visit interval of 5.43 months (*SD* = 1.47)—and blind parents (all mothers) who were the infants’ primary caregivers. Although the specific cause of the mothers’ visual impairment varied, all had experienced sight loss for more than 15 years and could not detect their infants’ eye gaze from a distance of ∼50 cm, based on their self-report (see the online supplemental materials for details about the mothers’ visual impairment and the family structure). Four additional recruited SIBP dyads were excluded from this study, as they did not attend assessments at both timepoints. All SIBP had undergone routine eye checks at or soon after birth and the parents were not aware of any sight problems in the infants, with the exception of one SIBP who was diagnosed with retinoblastoma soon after birth. This infant had undergone therapy for this condition prior to study participation, by which time (i.e., infant age 8 months old) the retinoblastoma was in remission (and remained so at Time 2) and the family had been told that infant’s vision had not been affected.

Data for control participants were made available via the British Autism Study of Infant Siblings Network (BASIS: www.basisnetwork.org; e.g., Bedford et al., 2012; Elsabbagh et al., 2012, 2014), which shared video recordings for 28 sighted typically developing infants (17 females) of sighted parents (all mothers). Again, data were available across two timepoints, when infants were Aged 6–10 months (*M* = 8.32, *SD* = 0.92) and 12–16 months (*M* = 14.69, *SD* = 1.01), with mean between-visit interval 6.37 months (*SD* = 0.77).

### Interaction Sampling and Coding Procedure

For the PCI sample, parent-child dyads were seated on a picnic mat in the assessment room, and provided with a small set of age-appropriate toys. Mothers were asked to play with their children as they would usually do at home, making use of the toys if desired. The experimenter left the dyad to play alone for 10 min, capturing footage via a remote video recording system. The SCI sample was drawn from video footage of infants interacting with a sighted, unfamiliar female researcher (one of 6 members of our research center) within a semistructured play-based assessment; the Autism Observation Scale for Infants (AOSI; Bryson, Zwaigenbaum, McDermott, Rombough, & Brian, 2008). Developed as a standardized behavior sample from which to observe social-communication and other behaviors in 6- to 18-month-olds at risk of developing ASD, the AOSI includes presses to elicit specific infant behaviors (e.g., the ability to track moving objects, to imitate actions, to respond to name call, etc.) and two 3–5 min periods during which the examiner engages the child in free play with standard age-appropriate toys. The aim of these free-play periods was to observe infant’s referential behavior, spontaneous vocalizations, and spontaneous actions directed at the toys or at the adult. We therefore used the AOSI free-play periods as naturalistic samples from which to code infant communicative behavior with an unfamiliar, sighted adult. Experimenters were aware of the infants’ group membership, but naive to the current study hypotheses. When interacting with an infant, the experimenter did not use a script but she prompted the infant to explore the toys provided, and responded to the infant’s vocalizations and behaviors directed at her.

The toys used in the SCI were different from those used in the PCI, as was the setup with infants seated on the floor with their parents for PCI, and on their parents’ lap across the table from the experimenter for the SCI. For each of the PCI and SCI, the setup and available toys were identical for all participants.

We coded infants’ communicative acts during the first 6 min of each interaction sample—PCI free play with the blind or sighted parent, and SCI free play with the unfamiliar sighted examiner—using aspects of the social-communication coding protocol of Clifford et al. (2010). Each infant communication act was assigned a specific function (i.e., initiation or response) and one or more forms (i.e., vocalization, action, and face gaze; see average scores in Table 1). An act was classified as an initiation if the infant’s communication behavior was not in direct response to a preceding adult behavior, and as a response when it followed on from something the adult had just said or done. The form of each act was classified as a vocalization when either a nonverbal vocalization, word approximation, or speech was used, as an action when there was some communicative movement of an object (e.g., holding something up to show it) or communicative use of the infant’s own body (e.g., reaching toward an object), and as face gaze when the infant looked toward the adult’s face or made a three-point gaze shift between the adult’s face and an object. Other more specific communicative forms were coded (e.g., pointing, giving/showing, head nodding/shaking, and following gaze), but these presented infrequently during the interaction samples for infants of this age and so were excluded from further analyses. Behavior combinations such as a vocalization accompanied by face gaze were coded as having only one communicative function but multiple communicative forms.[Table-anchor tbl1]

PCI coding from video footage commenced when the researcher left the parent and child to play alone and continued for 6 min. SCI coding from video footage commenced when the researcher placed the free-play toys on the table in front of the infant, and ended after 6 min (pausing when the researcher removed the toys at the end of the first AOSI free-play episode, and resuming when she returned these to the table for the second AOSI free-play episode).

To standardize the rates of communicative function codes across participants, we calculated an initiation-response index (IRI) by subtracting the number of responses from the number of initiations coded for each infant, and dividing this by the total number of communication acts. Hence, positive IRI values represent relatively more initiations and negative IRI values represent relatively more responses among an infant’s total communication acts. Similarly, the number of vocalizations, actions, and instances of face gaze were divided by the total number of infant communicative acts to obtain proportion measures of each communicative form (e.g., proportion vocalizations = number vocalizations/total communicative acts). As the communicative forms were not independent of one another, their sum could exceed 1. Total communication acts, IRI, and proportions of vocalizations, actions, and face gaze were then included in our key analyses.

### Evaluation of Interrater Agreement

Footage was coded by one of two raters, neither of whom was aware of the infants’ group status or age, or the study hypotheses. Interrater reliability was established by having both raters code a subset of clips, selected unsystematically, representing both the SIBP (*n* = 13 clips) and control groups (*n* = 30 clips) across both PCI (*n* = 27) and SCI (*n* = 16) contexts. Two-way mixed intraclass correlation coefficients (ICC_2,2_ with absolute agreement; see Trevethan, 2016) were used to evaluate interrater agreement across the key measures (see the Results section for a description of the measures). ICCs were adequate to excellent (Fleiss, 1986) for all the measures except for the IRI: total communication = .82 (ICC_2,1_ with *absolute* agreement); IRI = .62; proportion vocalizations = .91; proportion actions = .72; proportion face gaze = .87. The lower reliability score for the IRI may have been due to the fact that with very young infants it was more difficult to judge when they initiated communication than when they responded to the parent (ICC_2,1_ scores for Initiations = .45, and Responses = .77). ICC_2,1_ scores for the raw number of communicative forms are reported in the online supplemental materials. Note that the form of the ICC model changes for ICC_2,2,_ to ICC_2,1_ because the total number of communication acts and the raw number of communication forms were single measures, that were not averaged prior to the analysis.

## Results

We conducted a series of three-way analyses of variance (ANOVAs)—with group varying between participants and communication context and timepoint varying within participants.

**Figure 1 fig1:**
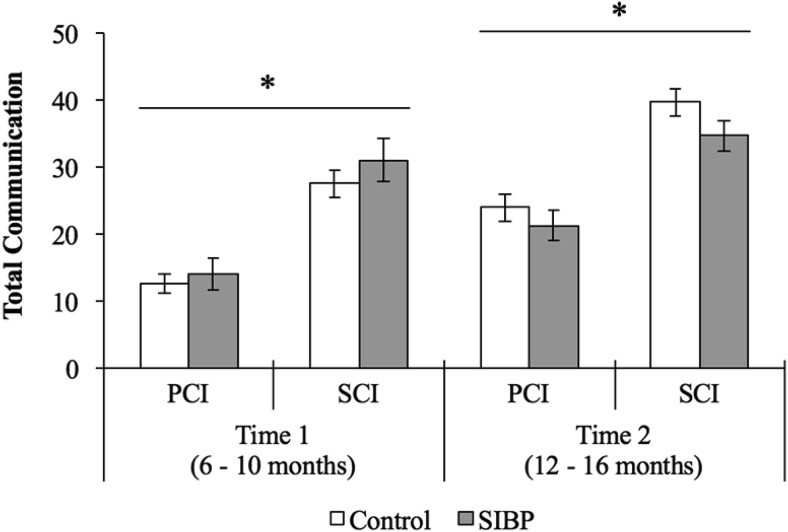
Total number of communication acts across groups, communication contexts, and timepoints. Error bars represent standard errors. PCI = parent-child interaction; SCI = stranger-child interaction; SIBP = sighted infants of blind parents. * *p* < .05.

The three-way ANOVA on total communication showed main effects of communication context (*F*(1, 40) = 76.81, *p* < .001, η_*p*_^2^ = .66) and timepoint (*F*(1, 40) = 36.36, *p* < .001, η_*p*_^2^ = .48), as infants communicated more often during SCI (*M* = 33.35, *SD* = 8.14) than PCI (*M* = 18.08, *SD* = 6.97), and more often at Time 2 (*M* = 30.56, *SD* = 6.68) than at Time 1 (*M* = 20.87, *SD* = 7.55). The latter main effect was qualified by a significant Timepoint × Group interaction term (*F*(1, 40) = 4.81, *p* = .034, η_*p*_^2^ = .11) such that controls used significantly more total communication acts at Time 2 (*M* = 31.84, *SD* = 7.07) than Time 1 (*M* = 20.05, *SD* = 6.65), *t*(27) = 7.96, *p* < .001, *d*_*z*_ = 1.50, whereas the differences in total communication acts between timepoints did not reach significance in SIBP (Time 2: *M* = 28.00, *SD* = 5.13; Time 1: *M* = 22.5, *SD* = 9.13), *t*(13) = 1.98, *p* = .07. The significance level for these post hoc tests and the ones reported hereafter was lowered to *p* = .025 after applying Bonferroni correction for multiple comparisons. Only those comparisons where *p* < .025 were reported as significant. Crucially, neither the main effect of group, *F*(1, 40) = .15, *p* = .70, nor the Communication Context × Group (*F*(1, 40) < .001, *p* = .98), nor the three-way interaction term, *F*(1, 40) = .65, *p* = .43 reached significance (Figure 1).[Fig-anchor fig1]

**Figure 2 fig2:**
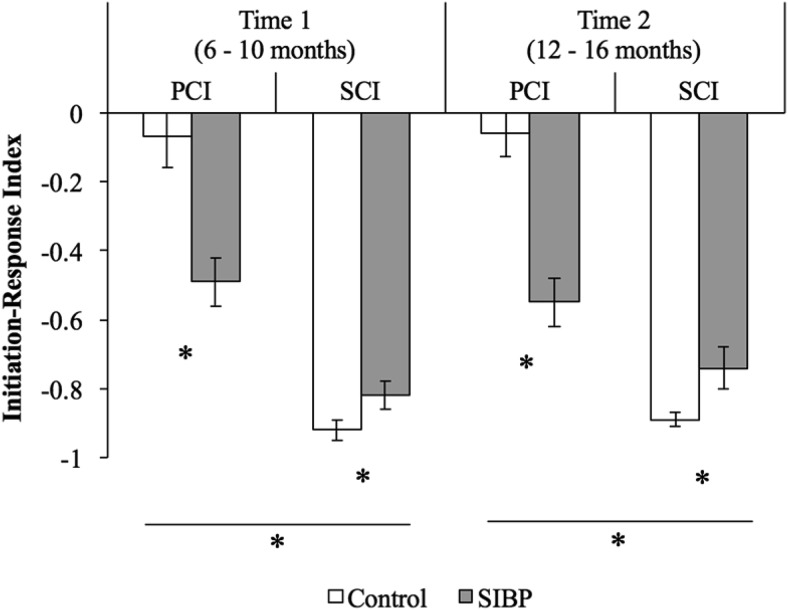
Initiation-response index [i.e., IRI = (initiations − responses)/(initiations + responses)] across groups, communication contexts, and timepoints. Negative values indicate more responses than initiations. Error bars represent standard errors. PCI = parent-child interaction; SCI = stranger-child interaction; SIBP = sighted infants of blind parents. * *p* < .05.

The mean IRI composite score was negative, overall, suggesting that the majority of infant communication functions were responses rather than initiations to the adult partners (Figure 2). However, results of the three-way ANOVA showed that IRI was modulated significantly by group membership and communication context. That is, there were significant main effects of group (*F*(1, 40) = 11.03, *p* = .002, η_*p*_^2^ = .22) and communication context (*F*(1, 40) = 131.01, *p* < .001, η_*p*_^2^ = .77). These effects were qualified, however, by a significant Group × Communication Context interaction term (*F*(1, 40) = 36.37, *p* < .001, η_*p*_^2^ = .48). Observed power was 90% for the significant main effect of group, 99% for the significant main effect of communication context, and 99% for the significant interaction. Follow-up analyses revealed that controls (*M* = −.07, *SD* = .31) initiated relatively more than SIBP (*M* = −.52, *SD* = .18) during PCI, *t*(40) = 5.07, *p* < .001, *d*_*s*_ = 1.77. Indeed, IRI of controls during PCI was very close to zero, implying a more balanced initiation and responses in this condition. By contrast, SIBP (*M* = −.78, *SD* = .15) initiated relatively more than controls (*M* = −.90, *SD* = .10) during SCI, *t*(19.28) = 2.86, *p* = .01, *d*_*s*_ = .94. No other main effects or interactions reached significance (Time-point effect, *F*(1, 40) = .108, *p* = .74; Group × Timepoint, *F*(1, 40) = .001, *p* = .98; Communication Context × Timepoint, *F*(1, 40) = .78, *p* = .38; three-way interaction, *F*(1, 40) = .39, *p* = .54).[Fig-anchor fig2]

**Figure 3 fig3:**
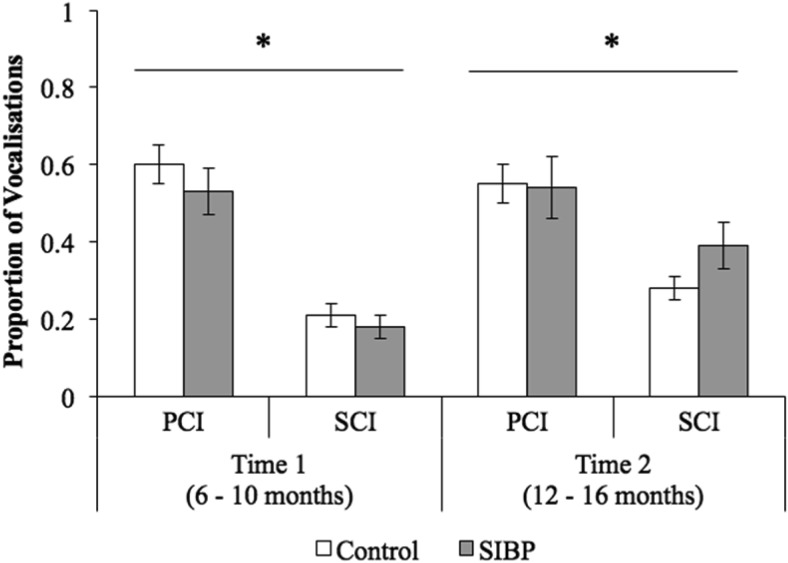
Proportion of vocalizations (i.e., number of vocalizations/total communication) across groups, communication contexts, and timepoints. Error bars represent standard errors. PCI = parent-child interaction; SCI = stranger-child interaction; SIBP = sighted infants of blind parents. * *p* < .05.

For vocalization, there was a significant main effect of communication context (*F*(1, 40) = 96.51, *p* < .001, η_*p*_^2^ = .71), with relatively more vocalization during PCI (*M* = .56, *SD* = .19) than SCI (*M* = .26, *SD* = .12; Figure 3). This was qualified by a significant Timepoint × Communication Context interaction term (*F*(1, 40) = 7.95, *p* = .007, η_*p*_^2^ = .17). Observed power was 99% for the significant main effect of communication context and 80% for the significant interaction. Follow-up analyses revealed that infants’ vocalizations increased between Time 1 (*M* = .20, *SD* = .16) and Time 2 (*M* = .32, *SD* = .19) during SCI, *t*(41) = 3.02, *p* = .004, *d*_*z*_ = .48, but not during PCI, *t*(41) = .61, *p* = .55 (*M*_Time1_ = .58, *SD*_Time1_ = .25; *M*_Time2_ = .55, *SD*_Time2_ = .25). No other main effects or interactions reached significance (group effect, *F*(1, 40) < .001, *p* = .99; timepoint effect, *F*(1, 40) = 2.57, *p* = .12; Group × Communication Context, *F*(1, 40) = 1.74, *p* = .19; Group × Timepoint, *F*(1, 40) = 1.69, *p* = .20; three-way interaction, *F*(1, 40) = .45, *p* = .51).[Fig-anchor fig3]

**Figure 4 fig4:**
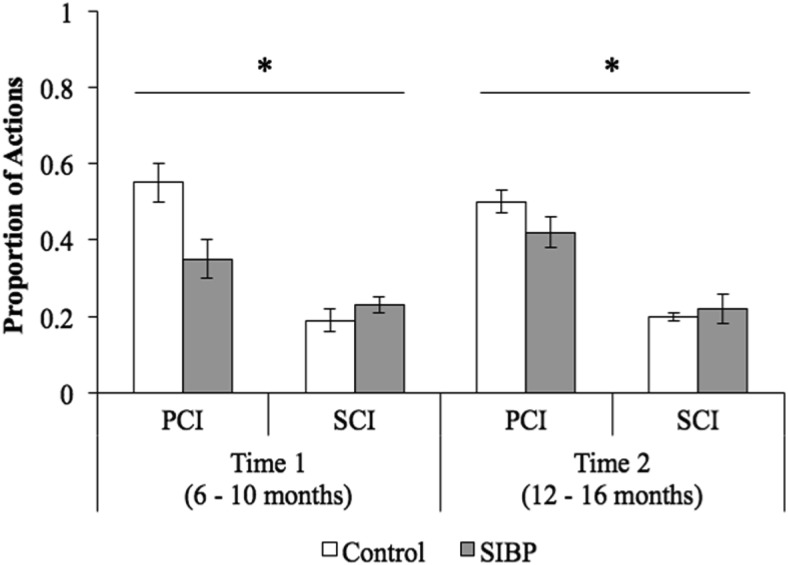
Proportion of action (i.e., number of actions/total communication) across groups, communication contexts, and timepoints. Error bars represent standard errors. PCI = parent-child interaction; SCI = stranger-child interaction; SIBP = sighted infants of blind parents. * *p* < .05.

A significant main effect of communication context for proportion of actions (*F*(1, 40) = 87.74, *p* < .001, η_*p*_^2^ = .69) reflected infants’ greater use of communicative actions during PCI (*M* = .48, *SD* = .17) compared with SCI (*M* = .21, *SD* = .08; Figure 4). This effect was qualified, however, by a significant Group × Communication Context interaction term (*F*(1, 40) = 10.04, *p* = .003, η_*p*_^2^ = .20). Observed power was 99% for the significant main effect of communication context and 87% for the significant interaction. Follow-up analyses revealed that, during PCI, SIBP (*M* = .38, *SD* = .13) used relatively fewer actions than controls (*M* = .52, *SD* = .17), *t*(40) = 2.72, *p* = .01, *d*_*s*_ = .93, whereas there was no such between-groups difference during SCI (SIBP: *M* = .22, *SD* = .08; control: *M* = .20, *SD* = .08), *t*(40) = .93, *p* = .36. No other main effects or interactions reached significance (group effect, *F*(1, 40) = 3.28, *p* = .08; timepoint effect, *F*(1, 40) = .009, *p* = .93; Group × Timepoint, *F*(1, 40) = .80, *p* = .38; Communication Context × Timepoint, *F*(1, 40) = .03, *p* = .86; three-way interaction, *F*(1, 40) = 1.84, *p* = .18).[Fig-anchor fig4]

**Figure 5 fig5:**
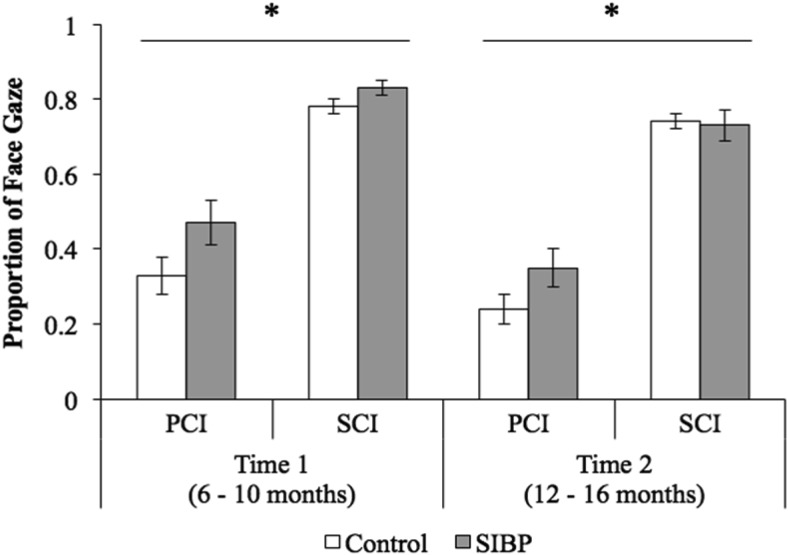
Proportion of face gaze (i.e., number of face gazes/total communication) across groups, communication contexts, and timepoints. Error bars represent standard errors. PCI = parent-child interaction; SCI = stranger-child interaction; SIBP = sighted infants of blind parents. *p < .05.

Finally, for proportion of face gaze, there were significant main effects of group (*F*(1, 40) = 4.60, *p* = .038, η_*p*_^2^ = .10), communication context (*F*(1, 40) = 235.11, *p* < .001, η_*p*_^2^ = .86), and timepoint (*F*(1, 40) = 12.73, *p* < .001, η_*p*_^2^ = .24). Observed power was 54% for the significant main effect of group, 99% for the significant main effect of communication context, and 93% for the significant main effect of time. These were such that SIBP used more face gaze (*M* = .60, *SD* = .09) than controls (*M* = .52, *SD* = .11), and all infants used more face gaze during SCI (*M* = .77, *SD* = .08) than PCI (*M* = .33, *SD* = .18), and at Time 1 (*M* = .59, *SD* = .14) compared with Time 2 (*M* = .51, *SD* = .13; Figure 5). The Communication Context × Group interaction approached significance, *F*(1, 40) = 3.622, *p* = .06, η_*p*_^2^ = .08, indicating marginally higher face gaze by SIBP (*M* = .41, *SD* = .15) compared with controls (*M* = .29, *SD* = .18) during PCI, *t*(40) = −2.28, *p* = .028, *d*_*s*_ = .73, compared with similar face gaze by infants in each group during SCI, *t*(40) = −.76, *p* = .45 (*M*_Control_ = .76, *SD*_Control_ = .09; *M*_SIBP_ = .78, *SD*_SIBP_ = .07). No other main effects or interactions reached significance (Group × Timepoint, *F*(1, 40) = .82, *p* = .37; Communication Context × Timepoint, *F*(1, 40) = .50, *p* = .49; three-way interaction, *F*(1, 40) = .08, *p* = .78).[Fig-anchor fig5]

## Discussion

This study represents a unique investigation of the communication behavior of SIBP, adopting a prospective follow-up design to examine interaction with both a blind parent and a sighted unfamiliar adult. We examined various aspects of infant communicative behavior—including both the function of communication acts and various forms of signaling these to the partner (i.e., via vocalization, action, and face gaze)—and found significant interactions between child group and social partner for some of these. Specifically, when they interacted with their blind parents, compared with control infants interacting with their own sighted parents, SIBP showed marked differences in both the function and the form of communication including using relatively more responses than initiations, and fewer communicative actions. By contrast, during interaction with a sighted unfamiliar adult, SIBP initiated relatively more than controls, with both groups using similar levels of communicative actions. A similar trend was observed for face gaze, where SIBP showed more face gaze than controls during interaction with their parents, but with no between-groups differences during interaction with a sighted stranger. Interestingly, both groups used similar levels of vocalizations, and vocalized more during the interaction with the parent than with a sighted stranger, and more at Time 2 than at Time 1. The results suggest that SIBP are flexibly and adaptively switching the style of their communication when with blind parents versus a sighted experimenter. This is consistent with the prediction derived from the interactive specialization model (Johnson, 2011), which hypothesizes that infants develop optimized communication behavior adaptive to the given communicative context. By contrast, it is inconsistent with the prediction derived from the affective learning viewpoint, which hypothesizes that infants learn the reward value of communication behavior through interaction with parents/caregivers and generalize this to other communicative contexts.

The directions of group differences in both the function and the form of communication are also informative, and somewhat counterintuitive. As for communicative function, SIBP responded more toward their parents than did controls, but initiated relatively more (or rather, responded relatively less) toward the sighted experimenter than did controls. This might suggest that SIBP have acquired skills to more effectively (or frequently) initiate communication to compensate for their parents’ difficulty to notice a visual form of communication. It may also be that this between-groups difference during PCI simply reflects a stronger tendency for initiated communication by blind (compared with sighted) parents—hence eliciting relatively more responses by their infants. However, this latter interpretation cannot account for the group differences also observed in communicative functions during the SCI condition (i.e., SIBP initiated relatively more than controls), in which both groups of infants were communicating with unfamiliar sighted adults.

As for the form of communication, SIBP used fewer communicative actions than controls, only when interacting with their parents, suggesting that SIBP also flexibly change the channels of communication depending on their communicative partner. It seems rational not to use actions—such as showing or reaching for an object—when these cues are less likely to be picked up by their blind parents. However, these results also showed that SIBP used a similar amount of these actions when they interacted with sighted communicative partner, suggesting that they can still use this channel of communication when it is efficient. In addition, overall higher use of face gaze by SIBP—particularly during interaction with their blind parents—may seem inconsistent with a previous study (Chiesa et al., 2015) which found shorter overall face gaze in SIBP. Possibly, this discrepancy is due to the adoption of different coding schemes. We coded the frequency of each form used in successful communication events, whereas Chiesa et al. (2015) coded the total frequency of each behavior during an observation period regardless of whether or not behaviors lead to successful communicative exchanges. Thus, it is possible that SIBP overall spend less time attending to parents’ faces, but efficiently respond to parental communicative bids with face gaze.

Methodological differences between studies may also explain the apparent contradiction between the results of the current study and those of our recently reported eye-tracking studies (Senju et al., 2015). Senju et al. (2015) found that SIBP and controls differ in terms of their gaze following behavior and face scanning pattern. Specifically, when presented with video clips of a female actress which looks directly toward the infant and then gazes at one of two objects in front of her, SIBP and controls follow equally frequently the gaze of actress to the object, but SIBP look for a shorter period of time at the gazed-at object that controls do. On the contrary, when watching a silent video of a dynamic female face, SIBP look more at the mouth than at the eyes area, whereas controls show the opposite face-scanning pattern, looking more at the eyes than at the mouth. The findings reported in the current paper, in contrast, are based on successful communication bids between infants and adults, and quantify different forms of communication among which is the proportion of looks to the adult’s face, irrespective of the part of the face attended to. In fact, given the interaction setup in the current study, it would be very difficult for us to report which part of the adults’ face infants gazed at when communicating. We therefore cannot rule out that the face-scanning pattern observed in the SIBP group by Senju et al. (2015) is specific to certain communication partners. Interestingly, Senju et al. (2015) found that SIBP and controls spent similar periods of time gazing to the dynamic female face. In the current study, we did not find a group difference in the proportion of face gaze in the SCI, but we did find a group difference in the PCI, suggesting that SIBP infants are adaptively changing their face-scanning behavior depending on whom they are interacting with. However, due to the low observed power for this statistical analysis, this result should be interpreted with caution. Further sufficiently powered follow-up researches will be informative to explore this interesting trend of the use of face gaze during communication in SIBP.

The longitudinal design of the study allowed us to also analyze developmental change from the latter half of the first year to the first half of the second year of the infants’ lives. The results showed almost no group differences in the developmental trajectory of functional communication or the forms used to signal these, with the exception of a main effect of reduced face gaze, and a specific increase in vocalizations toward a stranger, over time. Crucially, all of the between-groups differences we observed showed stability across Time 1 and Time 2 behavior samples, suggesting that SIBP acquired this partner-specific characteristic mode of communication early, and at least by 6 to 10 months of age.

Limitations in the current study arise from the difficulty in recruiting this hard-to-reach population and conducting assessments in a controlled environment. First, we could not fully match the communicative context between PCI and SCI, mainly because the video footage for SCI were taken from another semistructured behavioral assessment which might have contributed to some of the observed main effects of communication context for the function and form of infant communication behaviors. Thus, interpretation of these main effects needs to be treated with caution. However, this does not confound our observed between-groups differences, as both groups of infants participated in the same communicative context for each of PCI and SCI. Second, we did not code the adults’ communication behavior and cannot definitively say whether this was the same or different across groups. This could have affected the proportion of initiations and responses made by the infants, but it is less likely to have altered the proportions of forms of infant communication acts. Third, the reliability coefficient for the IRI, one of the measures on which we find differences between groups across both communication contexts, can be classified only as fair to good (Fleiss, 1986). This was mainly due to the fact that the IRI was computed as a function of raw number of initiations and responses, and that two raters found it more difficult to judge initiations than responses in young infants (see the Method section). In light of this fact, efforts should be made in future work to improve reliability on the function of communication acts in young infants either through better camera angle and higher video quality, or through double coding and consensus among raters on all the video clips coded. Fourth, despite being the largest sample reported for a study of this kind to date, power remains limited to detect small, but potentially developmentally important effects as statistically significant. Further replication studies, and/or follow-up studies with larger samples will be beneficial to test the robustness of the findings reported here, especially to further examine the effect of variability in social experience within the SIBP group (see the online supplemental materials for further analyses and discussions). Finally, we do not yet know whether the current findings are specific to SIBP or common to other populations who experience different forms of PCI, such as hearing infants of deaf parents. Future studies with more variable target populations will help us understand the specificity and generalizability of the unique communication behavior found in SIBP.

To conclude, the current research is the first to demonstrate that SIBP flexibly change their communication behaviors when interacting with their blind parents versus sighted unfamiliar adults. Such a capacity could relate to the advanced overall development reported in this population during the first year of life (Senju et al., 2015). The results highlight the plasticity inherent in the early development of human communicative skill, which enables infants to optimize their communication behaviors within the individual social environment.

## Supplementary Material

10.1037/dev0000564.supp

## Figures and Tables

**Table 1 tbl1:** Mean (SD) Number of Initiations, Responses, Vocalizations, Actions, and Face Gazes Across Groups, Timepoints, and Communication Contexts

	Initiations				Responses				Vocalizations				Actions				Face gazes			
	Time 1		Time 2		Time 1		Time 2		Time 1		Time 2		Time 1		Time 2		Time 1		Time 2	
Group	PCI	SCI	PCI	SCI	PCI	SCI	PCI	SCI	PCI	SCI	PCI	SCI	PCI	SCI	PCI	SCI	PCI	SCI	PCI	SCI
CTRL	5.61 (3.71)	.93 (1.51)	10.43 (4.60)	1.96 (1.50)	7.00 (4.97)	26.57 (11.02)	13.57 (8.13)	37.71 (10.99)	7.29 (5.32)	5.39 (4.69)	13.46 (8.27)	11.57 (8.29)	6.43 (4.37)	5.61 (4.71)	12.04 (6.53)	8.04 (3.76)	4.57 (4.64)	21.57 (9.29)	5.86 (4 .41)	29.29 (9.38)
SIBP	3.36 (2.34)	3.14 (3.11)	5.14 (4.02)	4.43 (3.74)	10.64 (7.29)	27.86 (10.61)	16.07 (6.26)	30.29 (8.72)	6.93 (3.45)	6.64 (5.76)	13.00 (9.77)	13.43 (8.51)	5.64 (5.17)	7.21 (4.28)	9.07 (5.03)	8.00 (5.53)	6.57 (5.23)	25.36 (8.75)	7.71 (5.01)	25.36 (8.43)
*Note.* PCI = parent-child interaction; SCI = stranger-child interaction; CTRL = control; SIBP = sighted infants of blind parents.																				

## References

[c1] Adamson L., Als H., Tronick E., & Brazelton T. B. (1977). The development of social reciprocity between a sighted infant and her blind parents. A case study. Journal of the American Academy of Child Psychiatry, 16, 194–207. 10.1016/S0002-7138(09)60036-4 874214

[c2] Bakeman R., & Adamson L. B. (1984). Coordinating attention to people and objects in mother-infant and peer-infant interaction. Child Development, 55, 1278–1289. 10.2307/1129997 6488956

[c3] Bedford R., Elsabbagh M., Gliga T., Pickles A., Senju A., Charman T., . . . the BASIS team (2012). Precursors to social and communication difficulties in infants at-risk for autism: Gaze following and attentional engagement. Journal of Autism and Developmental Disorders, 42, 2208–2218. 10.1007/s10803-012-1450-y 22278030

[c4] Bornstein M. H., Putnick D. L., Cote L. R., Haynes O. M., & Suwalsky J. T. D. (2015). Mother-infant contingent vocalizations in 11 countries. Psychological Science, 26, 1272–1284. 10.1177/0956797615586796 26133571 PMC4529355

[c5] Bryson S. E., Zwaigenbaum L., McDermott C., Rombough V., & Brian J. (2008). The Autism Observation Scale for Infants: Scale development and reliability data. Journal of Autism and Developmental Disorders, 38, 731–738. 10.1007/s10803-007-0440-y 17874180

[c6] Chiesa S., Galati D., & Schmidt S. (2015). Communicative interactions between visually impaired mothers and their sighted children: Analysis of gaze, facial expressions, voice and physical contacts. Child: Care, Health and Development, 41, 1040–1046. 10.1111/cch.12274 26250608

[c7] Clifford S., Hudry K., Brown L., Pasco G., & Charman T. (2010). The Modified-Classroom Observation Schedule to Measure Intentional Communication (M-COSMIC): Evaluation of reliability and validity. Research in Autism Spectrum Disorders, 4, 509–525. 10.1016/j.rasd.2009.11.008

[c8] Collis G. M., & Bryant C. A. (1981). Interactions between blind parents and their young children. Child: Care, Health and Development, 7, 41–50. 10.1111/j.1365-2214.1981.tb00819.x 7214669

[c9] Corkum V., & Moore C. (1995). Development of joint visual attention in infants In Moore C. & Dunham P. J. (Eds.), Joint attention: Its origins and role in development (pp. 61–83). Hillsdale, NJ: Erlbaum.

[c10] Csibra G. (2003). Teleological and referential understanding of action in infancy. Philosophical Transactions of the Royal Society of London Series B, Biological Sciences, 358, 447–458. 10.1098/rstb.2002.1235 12689372 PMC1693135

[c11] Dawson G., Webb S. J., & McPartland J. (2005). Understanding the nature of face processing impairment in autism: Insights from behavioral and electrophysiological studies. Developmental Neuropsychology, 27, 403–424. 10.1207/s15326942dn2703_6 15843104

[c12] DeCasper A. J., & Fifer W. P. (1980). Of human bonding: Newborns prefer their mothers’ voices. Science, 208, 1174–1176. 10.1126/science.7375928 7375928

[c13] Elman J. L., Karmiloff-Smith A., Bates E., Johnson M. H., Parisi D., & Plunkett K. (1996). Rethinking innateness: A connectionist perspective on development. Cambridge, MA: MIT Press.

[c14] Elsabbagh M., Bedford R., Senju A., Charman T., Pickles A., Johnson M. H., . . . BASIS Team (2014). What you see is what you get: Contextual modulation of face scanning in typical and atypical development. Social Cognitive and Affective Neuroscience, 9, 538–543. 10.1093/scan/nst012 23386743 PMC3989131

[c15] Elsabbagh M., Mercure E., Hudry K., Chandler S., Pasco G., Charman T., . . . the BASIS Team (2012). Infant neural sensitivity to dynamic eye gaze is associated with later emerging autism. Current Biology, 22, 338–342. 10.1016/j.cub.2011.12.056 22285033 PMC3314921

[c16] Farroni T., Csibra G., Simion F., & Johnson M. H. (2002). Eye contact detection in humans from birth. Proceedings of the National Academy of Sciences of the United States of America, 99, 9602–9605. 10.1073/pnas.152159999 12082186 PMC123187

[c17] Fleiss J. (1986). The design and analysis of clinical experiments. New York, NY: Wiley.

[c18] Goldstein M. H., & West M. J. (1999). Consistent responses of human mothers to prelinguistic infants: The effect of prelinguistic repertoire size. Journal of Comparative Psychology, 113, 52–58. 10.1037/0735-7036.113.1.52 10098268

[c19] Grelotti D. J., Gauthier I., & Schultz R. T. (2002). Social interest and the development of cortical face specialization: What autism teaches us about face processing. Developmental Psychobiology, 40, 213–225. 10.1002/dev.10028 11891634

[c20] Gros-Louis J., West M. J., & King A. P. (2016). The influence of interactive context on prelinguistic vocalizations and maternal responses. Language Learning and Development, 12, 280–294. 10.1080/15475441.2015.1053563

[c21] Jaswal V. K., & Fernald A. (2002). Learning to communicate In Lewis M. & Slater A. (Eds.), Introduction to infant development (pp. 244–265). Oxford, UK: Oxford University Press.

[c22] Johnson M. H. (2011). Interactive specialization: A domain-general framework for human functional brain development? Developmental Cognitive Neuroscience, 1, 7–21. 10.1016/j.dcn.2010.07.003 22436416 PMC6987575

[c24] Rattray J., & Zeedyk M. S. (2005). Early communication in dyads with visual impairment. Infant and Child Development, 14, 287–309. 10.1002/icd.397

[c25] Senju A., Tucker L. A., Pasco G., Hudry K., Elsabbagh M., Charman T., & Johnson M. H. (2013). The importance of the eyes: Communication skills in infants of blind parents. Proceedings of the Royal Society of London Series B: Biological Sciences, 280, 20130436 10.1098/rspb.2013.0436 23576790 PMC3652463

[c26] Senju A., Vernetti A., Ganea N., Hudry K., Tucker L., Charman T., & Johnson M. H. (2015). Early social experience affects the development of eye gaze processing. Current Biology, 25, 3086–3091. 10.1016/j.cub.2015.10.019 26752077 PMC4683081

[c27] Sommerville J. A., Woodward A. L., & Needham A. (2005). Action experience alters 3-month-old infants’ perception of others’ actions. Cognition, 96, B1–B11. 10.1016/j.cognition.2004.07.004 15833301 PMC3908452

[c28] Tadić V., Pring L., & Dale N. (2009). Attentional processes in young children with congenital visual impairment. British Journal of Developmental Psychology, 27, 311–330. 10.1348/026151008X310210 19998534

[c29] Tamis-LeMonda C. S., Kuchirko Y., & Tafuro L. (2013). From action to interaction: Infant object exploration and mothers’ contingent responsiveness. IEEE Transactions on Autonomous Mental Development, 5, 202–209. 10.1109/TAMD.2013.2269905

[c30] Trevethan R. (2016). Intraclass correlation coefficients: Clearing the air, extending some cautions, and making some requests. Health Services and Outcomes Research Methodology, 17, 127–143.

[c31] Wan M. W., Green J., Elsabbagh M., Johnson M., Charman T., & Plummer F., & the BASIS Team (2013). Quality of interaction between at-risk infants and caregiver at 12–15 months is associated with 3-year autism outcome. Journal of Child Psychology and Psychiatry, 54, 763–771. 10.1111/jcpp.12032 23227853

