# Supplementary material for: Development of Adaptive Communication Skills in Infants of Blind Parents
Source: Dev Psychol. 2018 Oct 18;54(12):2265–73. doi: 10.1037/dev0000564 (PMC6254470; doi:10.1037/dev0000564)
Supplement: Supplementary file 1 [file Supplemental-Materials.docx]

Supplementary Information

- Three-way ANOVA on the raw number of initiations – with Group varying between participants and Communication context and Time-point varying within participants.

The three-way ANOVA on the raw number of initiations showed main effects of Communication context (*F*(1, 40) = 68.30, *p* < .001, *η_p_^2^* = .63) and Time-point (*F*(1, 40) = 17.73, *p* < .001, *η_p_^2^* = .31). Infants initiated more often during PCI (*M* = 6.76, *SD* = 3.26) than SCI (*M* = 2.23, *SD* = 2.02), and more often at Time 2 (*M* = 5.73, *SD* = 2.96) than at Time 1 (*M* = 3.26, *SD* = 1.93). These two main effects were qualified by a significant Communication context x Time-point interaction term (*F*(1, 40) = 4.32, *p* = .044, *η_p_^2^* = .10) such that the number of initiations during SCI increased slightly at Time 2 (*M*  = 2.79, *SD* = 2.70) compared to Time 1(*M* = 1.67, *SD* = 2.39), *t*(41) = 2.33, *p* < .025, *d_z_* = .36, but it almost doubled between visits for the PCI (Time 1: *M* = 4.86, *SD* = 3.45; Time 2: *M* = 8.67, *SD* = 5.04), *t*(41) = 4.35, *p* < .001, *d_z_* = .67. Furthermore, there was a significant Group x Communication context interaction (*F*(1, 40) = 51.46, *p* < .001, *η_p_^2^* = .56). Thus during PCI, Controls (*M* = 8.02, *SD* = 2.94) initiated more than SIBP (*M* = 4.25, *SD* = 2.30), *t*(40) = 4.19, *p* < .001, *d_s_* = 1.43, while during SCI, Controls (*M* = 1.45, *SD* = 1.16) initiated less than SIBP (*M* = 3.79, *SD* = 2.48), *t*(15.90) = 3.35, *p* = .004, *d_s_* = 1.21. No other main effects or interactions reached significance (Group, *F*(1, 40) = 1.35, *p* = .25; Group x Time-point, *F*(1, 40) = 1.73, *p* = .20; three-way interaction, *F*(1, 40) = 2.54, *p* = .12).


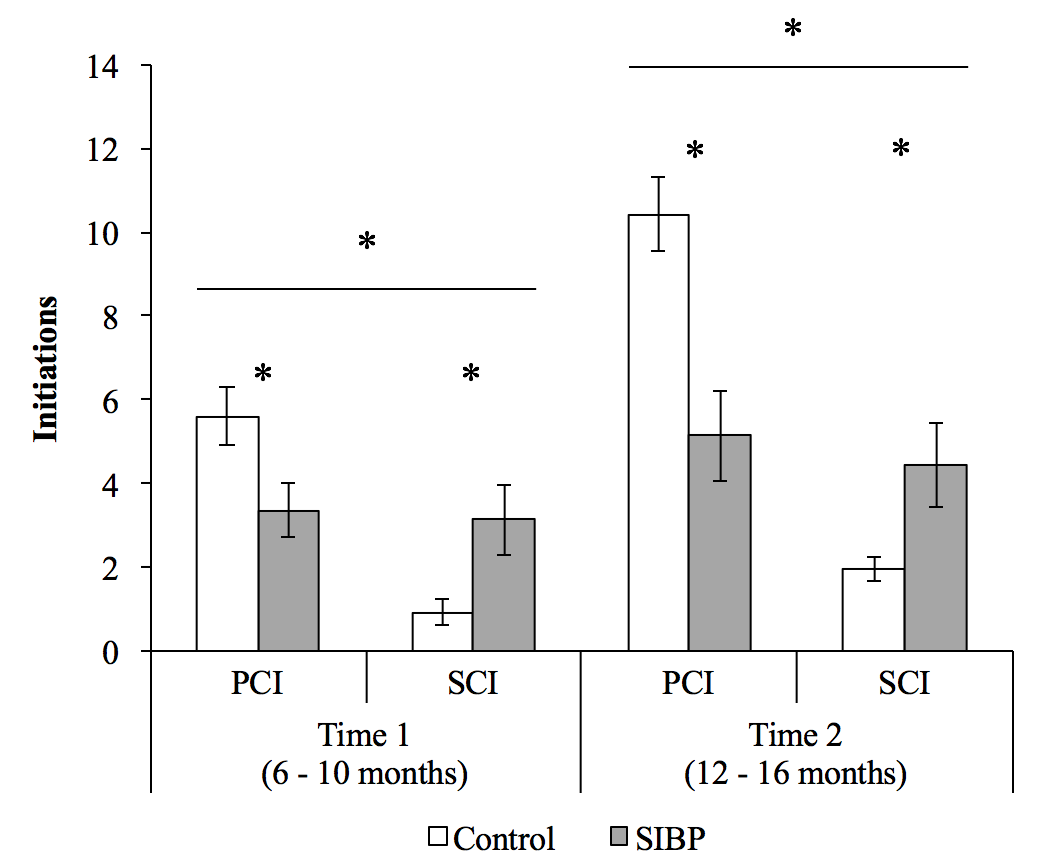


*Figure S1*. Number of initiations across groups, communication contexts, and time-points. Error bars: *SE*. * indicates *p* < .05.

- Three-way ANOVA on the raw number of responses

A three-way ANOVA on the raw number of responses revealed a main effect of Communication context (*F*(1, 40) = 131.59, *p* < .001, *η_p_^2^* = .77) and Time-point (*F*(1, 40) = 23.20, *p* < .001, *η_p_^2^* = .37). Across both groups, infants responded more during the SCI (*M* = 31.12, *SD* = 8.25) than the PCI (*M* = 11.31, *SD* = 5.53), and more at Time 2 (*M* = 24.82, *SD* = 6.09) than at Time 1 (*M* = 17.61, *SD* = 6.58). The interactions Group x Communication context (*F*(1, 40) = 3.52, *p* = .068, *η_p_^2^* = .08) and Group x Time-point (*F*(1, 40) = 3.45, *p* = .071, *η_p_^2^* = .08) missed significance. No other main effects or interactions were found (Group, *F*(1, 40) < .001, *p* = 1.00; Communication context x Time-point, *F*(1, 40) = .10, *p* = .752; three-way interaction, *F*(1, 40) = 2.36, *p* = .133).


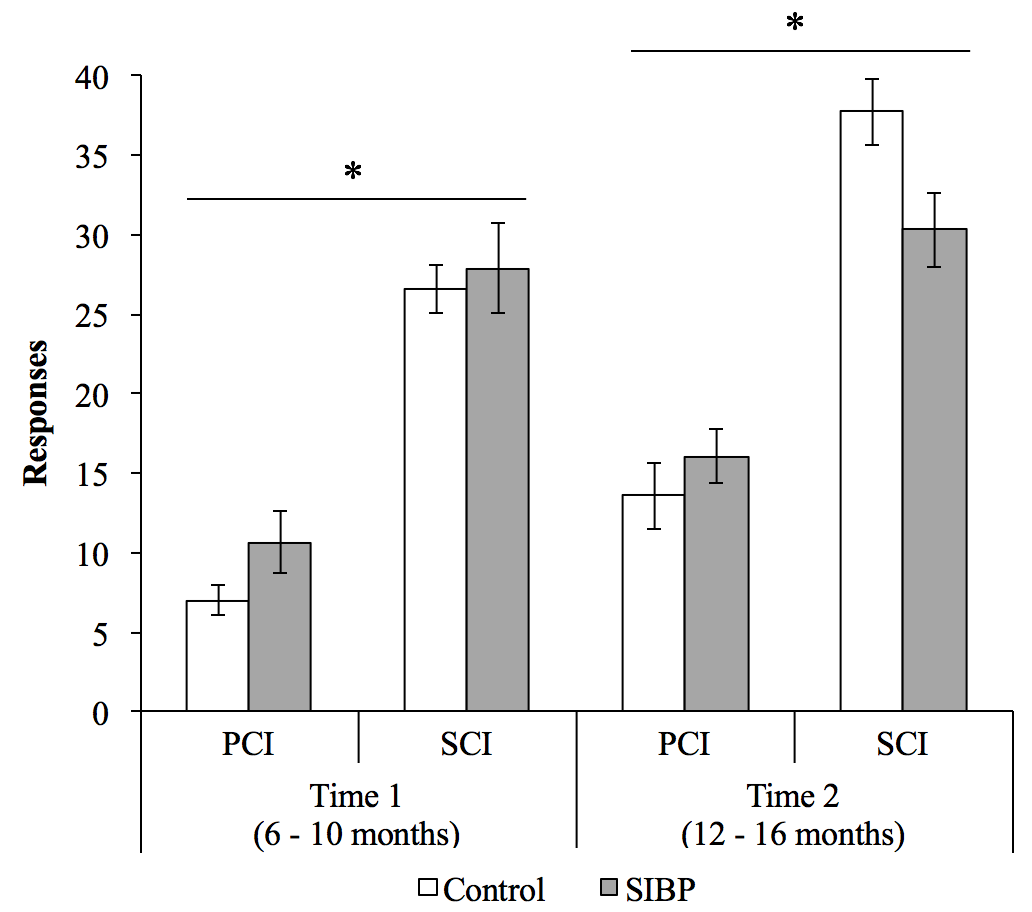


*Figure S2*. Number of responses across groups, communication contexts, and time-points. Error bars: *SE*. * indicates *p* < .05.

- Intra-class correlations for the raw number of vocalisations, actions, face gazes

Two-way mixed intra-class correlation coefficients (ICC_2,1_ with *absolute* agreement) were performed on the number of the commutative forms, thus: Vocalisations = .75, Actions = .60, Face Gaze = .90.

- Exploratory analyses for the impact of within-group diversity in the level of parent's visual impairment and the experience of interaction with other sighted people.

In the SIBP group, the main caregiver (i.e. the mother) of all 14 infants was visually impaired. The level of visual impairment varied between having no light perception (N = 5), and being able to detect the infant’s head orientation from ~50 cm distance (N = 7). The rest of the mothers (N = 2) had light perception, but could not detect the infant’s head orientation. Based on self-report, none of the mothers could detect their baby’s eye gaze direction from ~50 cm distance (which is approximately an arm-length away). We split the SIBP group for those with parents who could detect the infant's head orientation and those with parents who could not. In the PCI, infants whose mothers could detect head orientation tended to have a higher proportion of face gaze (*M* = .51, *SD* = .05) than those infants whose mothers could not (*M* = .31, *SD* = .16; *t*(6.93) = 3.05, p = .019; *d_s_* = 1.69). In the SCI, infants whose mothers could detect head orientation tended to vocalise more (*M* = .34, *SD* = .10) than infants whose mothers could not detect head orientation (*M* = 23, *SD* = .09; *t*(12) = 2.29, p = .04; *d_s_* = 1.16). However, cautions should be taken to interpret these results, because none of these differences would survive when Bonferroni correction was applied. No other differences reached significance even at the uncorrected level.

The family structure also varied across infants. The parents of just under half of the infants (N = 6) both experienced visual impairment. While for the rest of the infants (N = 8), only the mother was visually impaired. Furthermore, only half of the infants (N = 7) had an older sibling. The older sibling was either sighted (N = 5), or visually impaired (N = 2). Although we did not have an exact measure time estimate of how long infants spent only with visually impaired adults, or only with sighted adults, all the families reported that infants encountered sighted adults on a daily basis, for brief periods of time (while attending baby classes, going shopping, or visiting relatives or family friends).

To assess whether family structure affected SIBPs’ communication pattern, we split the SIBP group into infants whose both parents were visually impaired (N = 6), and infants whose mother was visually impaired, but the father was sighted (N = 8). *T*-tests showed that, in the PCI, there was no difference between groups on the total communication score, IRI, proportion of vocalisations, proportion of actions, and proportion of face gaze (all *t* < 1.72, *p* > .112). Similarly, in the SCI, the groups did not differ significantly on total communication, proportion of vocalisations, actions, and face gaze (all *t* < 1.71, all *p* > .115), but they tended to differ on IRI: Infants whose both parents were visually impaired initiated slightly more (*M* = -.68, *SD* = .17) than those infants whose mother was visually impaired, but not the father (*M* = -.85, *SD* = .08; *t*(12) = 2.36, *p* = .036, *d_s_* = .86). This pattern of results suggests that SIBPs’ communication pattern with their mother is not related to their level of exposure to sighted adults (i.e. higher if they have a sighted father, lower if both the father and the mother are visually impaired). On the other hand, when interacting with sighted adults, infants whose both parents were blind, tended to initiate communication more often with sighted strangers, which is in line with the main finding that experience with blind caregivers could result in tendency to initiate more when communicating with sighted strangers. However, this result should be interpreted with caution, as the significant difference would not survive if Bonferroni correction was applied.

Having an older sibling also seemed to influence how SIBP infants communicated with their visually impaired mother: Infants who had an older sighted sibling (N = 5) used more actions when communicating with their mothers (*M* = .48, *SD* = .10), than infants who did not have a sibling (N = 7; *M* = .32, *SD* = .10; *t*(10) = 2.70, *p* = .022, *d_s_* = 1.12), which might suggest that having an older sibling could reduce the effect of having a blind parent on the differences in the use of action during communication with blind parents. However, this result should be interpreted with caution, as the significant difference would not survive if Bonferroni correction was applied. No other differences were found between SIBP infants with and without an older sibling irrespective whether they interacted with their mother (all *t* < 1.65, all *p* > .130), or a sighted stranger (all *t* < 1.74, all *p* > .112), even at the uncorrected threshold for significance.

Some of the SIBP also attended nursery or had contact with a child-minder. To quantify the amount of experience that infants had with other sighted adults, parents were asked to indicate the number of hours that infants spent in day care per week, and the number of hours that infants spent seeing sighted adults per week (including day care hours). This was then averaged across the two Time-points.

The number of day care hours varied between 0 hours (N = 9) and 50 hours per week (N = 1). A correlational analysis was conducted between the number of hours infants spent in day care and the total communication score, IRI, proportion of vocalisations, actions, and face gaze in the PCI, and the SCI. None of the correlations was significant (all *r*(12) < .34, *p* > .23).

Similarly, no relationship was found between the number of hours that infants spent in the company of sighted adults (other adults than the father) and their total communication, IRI, proportion of vocalizations, actions, and face gaze in the PCI and SCI (all *r*(12) < .33, *p* > .25). This analysis suggests that experience with sighted adults, other than the father, does not impact significantly on SIBP infants’ communication pattern with adults.
